# Identification and verification of microtubule associated genes in lung adenocarcinoma

**DOI:** 10.1038/s41598-023-42985-3

**Published:** 2023-09-26

**Authors:** YuHui Wei, CaiZhen Yang, JinMei Wei, WenTao Li, YuanWen Qin, GuangNan Liu

**Affiliations:** Department of Respiratory and Critical Care, The Second Affiliated Hospital of Guangxi Medical University, Nanning, China

**Keywords:** Cancer, Computational biology and bioinformatics

## Abstract

Associated with high morbidity and mortality, lung adenocarcinoma (LUAD) is lacking in effective prognostic prediction and treatment. As chemotherapy drugs commonly used in clinics, microtubule-targeting agents (MTAs) are limited by high toxicity and drug resistance. This research aimed to analyze the expression profile of microtubule-associated genes (MAGs) in LUAD and explore their therapy efficiency and impact on prognosis. Key MAGs were identified as novel molecular targets for targeting microtubules. The LUAD project in The Cancer Genome Atlas (TCGA) database was used to identify differently expressed MAGs. On the one hand, a microtubule-related prognostic signature was constructed and validated, and its links with clinical characteristics and the immune microenvironment were analyzed. On the other hand, hub MAGs were obtained by a protein–protein interaction (PPI) network. Following the expression of hub MAGs, patients with LUAD were classified into two molecular subtypes. A comparison was made of the differences in half-maximal drug inhibitory concentration (IC50) and tumor mutational burden (TMB) between groups. In addition, the influence of MAGs on the anticancer efficacy of different therapies was explored. MAGs, which were included in both the prognosis signature and hub genes, were considered to have great value in prognosis and targeted therapy. They were identified by quantitative real-time polymerase chain reaction (qRT-PCR). A total of 154 differently expressed MAGs were discovered. For one thing, a microtubule-related prognostic signature based on 14 MAGs was created and identified in an external validation cohort. The prognostic signature was used as an independent prognostic factor. For another, 45 hub MAGs were obtained. In accordance with the expression profile of 45 MAGs, patients with LUAD were divided into two subtypes. Distinct differences were observed in TMB and IC50 values of popular chemotherapy and targeted drugs between subtypes. Finally, five genes were included in both the prognosis signature and hub genes, and identified by qRT-PCR. A microtubule-related prognosis signature that can serve as an independent prognostic factor was constructed. Microtubule subtype influenced the efficacy of different treatments and could be used to guide therapy selection. In this research, five key MAGs, including MYB proto-oncogene like 2 (MYBL2), nucleolar and spindle-associated protein 1 (NUSAP1), kinesin family member 4A (KIF4A), KIF15 and KIF20A, were verified and identified. They are promising biomarkers and therapeutic targets in LUAD.

## Introduction

Lung cancer has become one leading cause of cancer-related mortality^1^, and its most common subtype is lung adenocarcinoma (LUAD). Tumor-node-metastasis (TNM)-based staging is the most widely used strategy for predicting survival in the clinic. However, the prognosis of lung cancer differs substantially among patients of the same stage. Nowadays, surgery, radiotherapy, chemotherapy, immunotherapy and molecular-targeted therapy are the main treatments for LUAD. Multiple therapies bring new opportunities to patients with LUAD. Nevertheless, valid markers are needed to guide treatment selection. In addition, blockades still exist, including drug resistance^2^, big side effects^3^ and low response rates. Hence, effective biomarkers and therapeutic targets remain urgently needed by LUAD patients. It is necessary to further explore the molecular mechanisms of abnormal microtubule activities and provide evidence for the development of molecularly targeted drugs.

Microtubules which are hollow tubes assembled by tubulins play a crucial role in multiple cellular processes as parts of the cytoskeleton^4^. They have become one of the effective antitumor targets because tumor cell proliferation can be inhibited by interfering with microtubule assembly. Multiple microtubule-targeting agents (MTAs) have been synthesized and employed as first-line anticancer drugs like taxane and colchicine. They significantly prolong the survival of patients. The feasibility of microtubules as anti-tumor targets is unquestionable.

Many studies have been conducted on prognostic signatures like epithelial-mesenchymal transition (EMT)-related signatures^5^ and hypoxia-related signatures^6^. However, very little research has investigated the value of microtubules in prognosis and guiding treatment.

In this research, the expression and clinical significance of microtubule-associated genes (MAGs) in LUAD were explored systematically. The gene-expression profiling and clinical features of 489 LUAD samples were obtained from The Cancer Genome Atlas (TCGA) database. MAGs were found from the gene set enrichment analysis (GSEA) database. Based on differently expressed MAGs, a microtubule-related prognostic signature was constructed and identified in a validation cohort. The prognostic value of MAGs in LUAD was highlighted. Unlike general prognosis signatures, the hub genes of differently expressed MAGs were further identified. According to the expression levels of hub MAGs, 489 patients were divided into two groups. A comparison was made of tumor mutational burden (TMB) and the IC50 values of popular chemotherapy and targeted drugs between the two groups. In addition, their relationships with the tumor immune microenvironment were analyzed. MAGs, which were included in both the prognosis signature and hub genes, were considered to be related to both prognosis and chemotherapy sensitivity in LUAD and served as potential targets. Finally, five MAGs were verified in total by quantitative real-time polymerase chain reaction (qRT-PCR).

The value of MAGs in prognosis and guiding treatment was assessed to identify and verify key MAGs. It will provide a foundation for the establishment of biomarkers and the development of molecular MTAs.

## Materials and methods

### Data collection and process

Ribonucleic acid (RNA) sequencing profiles (including both fragments per kilobase of exonmodelper million mapped fragments (FPKM) and COUNTS values), somatic variation and accompanying clinical data were collected from the TCGA-LUAD project^7^ as training cohorts.

Transcript expression data were obtained from 594 samples and clinical information was obtained from 585 patients in total. After the exclusion of those without survival time, stage or metastasis, 489 LUAD samples with reasonably completed clinical information were selected for the next analysis. GSE50081, a validation set, was retrieved from the Gene Expression Omnibus (GEO) database^8^ using the package TCGA biolinks^9^.

To find microtubule-related ontology gene sets, “microtubule” was utilized as a search phrase in the Molecular Signatures Database (MSigDB)^10^. All the genes engaged in these pathways were regarded as MAGs.

### Enrichment analysis of differently expressed genes

The DESeq2 package^11^ was used for comparing expression profiles between tumor and normal samples. Genes with FC > 1.5 and p < 0.05 were viewed as differently expressed genes (DEGs). Up- and down-regulation genes were presented on the volcano map. GSEA was employed for the detection of differently activated biological processes using the clusterProfiler^12^ package. As reference gene sets, the biological processes ontology derived from the gene ontology resource were employed. The pathways whose adjusted p-values are under 0.05 were regarded as differently activated pathways.

### Construction of microtubule-associated prognostic signature

Differently expressed MAGs were defined by taking the intersection of DEGs and MAGs. Univariate and multivariate Cox regressions were performed to determine independent prognostic MAGs. After univariate Cox regression analysis, differently expressed MAGs were subjected to performing multivariate Cox regression. Independent prognostic genes were defined as multivariate analysis with P < 0.05. After that, the least absolute shrinkage and selection operator (LASSO), a machine learning approach to selecting variables in multicollinear variables, was employed to squeeze genes. Finally, a Cox proportional hazards model was created. The above procedures were carried out by use of the R package glmnet^13^. To ensure repeatability, the random number seed was set as 3. The best lambda value was obtained by cross-validation 1,000 times. At last, the best genes for the construction of the microtubule-associated prognostic signature were chosen. The signature was constructed using the following formula:

$$Riskscore={\sum }_{n=1}^{\infty }\left(\mathrm{Coefficient}(\mathrm{i}) *\mathrm{ Xi}\right)$$

The coefficient was presented from the weight of multivariate analysis. Time-dependent receiver operating characteristic (ROC) curves were utilized for evaluating the effectiveness of the signature and created by using the R package time ROC.

### Establishment and evaluation of a nomogram

For ease in clinical application, a nomogram combining multiple clinical indicators was established. The discrimination ability to predict survival outcomes was assessed by the concordance index (C index) and decision curve analysis (DCA). In addition, the calibration plots of 1, 3 and 5 years were created to testify uniformity power.

### Prognostic value of the signature

The model was used to derive and group the risk scores of each patient. The patients were separated into two groups based on their median risk scores. The Kaplan–Meier (KM) technique was used to assess the capability of categorizing death risks.

To further investigate the heterogeneity between the two groups, the differences in clinical features were compared. Clinical features included age, gender and stages T, N, M. T, N and M stand for original tumor diameter, distant metastasis and lymph node metastasis, respectively. The information containing letters TX, MX and NX was deemed unclear and not taken into account in statistical analysis.

### Immune landscape analysis

Immunotherapy is an important part of anti-cancer therapy. This research was aimed at comparing the immune microenvironment to see if any variation existed in the immune landscape. Cibersort and xcell were chosen as prediction algorithms because of quantifying immune infiltration based on different principles. Cibersort is a deconvolution-based algorithm, whereas xcell calculates the proportions of cells based on marker genes by single sample GSEA (ssGSEA)^14^. The proportion of 22 common immune cells was quantified by Cibersort, and helper T cells were quantified by xcell. The aforesaid algorithms were deployed on TIMER2.0^15^, an open-access portal that integrates multiple immune infiltration techniques.

Immune checkpoint inhibitors (ICIs) have been utilized as one of the main treatments for LUAD with satisfactory effect. The expression levels of programmed death protein 1 (PD-1), programmed death ligand 1 (PD-L1) and lymphocyte activation gene-3 (LAG3) were examined as common immune checkpoints, to predict the response of ICIs.

### External validation of the signature

The signature was validated by selecting GEO datasets as external cohorts. In the validation cohort, risk scores were calculated, and the patients were separated into low- and high-score groups. The prognostic value of the signature was examined by performing KM analysis and time-dependent ROC curves. Immune cell infiltration was also measured and used to investigate its links with the immune microenvironment. The procedures of these analyses were the same as those in training cohorts.

### Protein–protein interaction network

The STRING database is an integrative database for the consolidation and prediction of protein interaction. Based on the database, the correlations of differently expressed MAGs were described and then displayed as a protein–protein interaction (PPI) network in Cytoscape. Molecular complex detection (MCODE) is a kind of strong software for locating key subnetworks in a huge interactive network according to the relationship between edges and nodes. Subnetworks were screened by MCODE, and the genes found there were designated as hub MAGs.

### Identification of subgroups using consensus clustering

Based on hub MAGs, unsupervised clustering was performed to identify subgroups using the R package ConsensusClusterPlus. The Partitioning Around Medoids (PAM) algorithm was used as a clustering algorithm, and sampling was performed 1000 times with a sub-sampling ratio of 0.8. The seed for the random number generator was 123,456. The optimal K value was obtained while the cumulative distribution function (CDF) reached a near-maximal value and remained constant. Heatmaps were visually inspected to identify the optimal number of gene clusters. T-distributed stochastic neighbor embedding (tSNE) analysis was applied, to ensure the accuracy of classification. Then, survival outcomes between clusters were compared by using survival analysis.

### Comparison of biological pathways and therapeutic effect

Difference-in-difference and GSEA analyses described above were performed to probe into the biological function of hub MAGs. Then, TMB and IC50 were used as indicators to predict diverse therapeutic effects. TMB was defined to be the number of somatic mutations in every 1 Mb exon. Patients with higher TMB are more likely to benefit from immunotherapy. The R package maftools were used for analyzing somatic mutations and calculating the TMB of each patient. The IC50 value was used to evaluate and compare the response to a variety of treatments. Lower IC50 indicated higher drug sensitivity. The IC50 of commonly used drugs including targeted and chemotherapy drugs was estimated using R package pRRophetic.

### Identification of differently expressed hub MAGs

After the intersection of DEGs and hub MAGs was taken, five differentially expressed hub MAGs were obtained: nucleolar and spindle-associated protein 1 (NUSAP1), KIF15, MYBL2, KIF20A and KIF4A. Multiple immunohistochemistry results of proteins in various tissues were found in the Human Protein Atlas (HPA) database, which provided a wealth of resources for protein-level gene verification. Matching protein expression in normal lung and LUAD was downloaded and compared to verify the expression of differently expressed hub MAGs in LUAD.

### Cell culture

A549 and BEAS-2B were purchased from NEWGAINBIO company and cultured in an incubator with 5% carbon dioxide (CO_2_) at 37 °C. Then, 1640 (Gibco, the United States of America (USA)) with 10% fetal bovine serum (FBS; BI, China) was used as the culture medium of A549 cells. Dulbecc’s modified Eagle’s medium (DMEM, Gibco, USA) with 10% FBS (BI, China) was used as the culture medium of BEAS-2B cells.

### qRT-PCR

After more than 80% cell fusion rates, the cells were treated with TRIzol Reagent (Takara, Japanese) to isolate total RNA. Next, complementary deoxyribonucleic acid (cDNA) was synthesized by use of the PrimeScript™ RT reagent Kit with genomic DNA (gDNA) Eraser (Takara, Japanese) once RNA purity was determined. On the StepOnePlus real-time PCR system (StepOnePlus; Applied Biosystems), 2× RealStar Fast SYBR qPCR Mix (Genstar, China) was used to perform RT-qPCR. β-Actin (ACTB) was regarded as a reference gene to normalize gene expression. All specific primers were synthesized by Sangon Biotechnology (Shanghai, China).

### Statistical analysis

R 4.1.2 and GraphPad Prism version 8.0 (GraphPad Software, Inc., La Jolla, California (CA), USA) were used to perform all the above procedures. Wilcoxon rank-sum test was used to compare the differences between low- and high-score groups, including clinical characteristics, immune checkpoint expression, TMB and IC50. Survival curves were drawn by the KM analysis and then compared by the log-rank test. Student’s t-test was used to evaluate the differences in the expression levels of MAGs between A549 and BEAS-2B. The inspection level was set as 0.05.

### Ethics approval and consent to participate

Both TCGA and GEO are public databases. Users can download relevant data for free to conduct research and post relevant articles. Based on open-source data, this study required no ethical approval.

## Result

### Identification and enrichment analysis of DEGs

**Figure 1:**
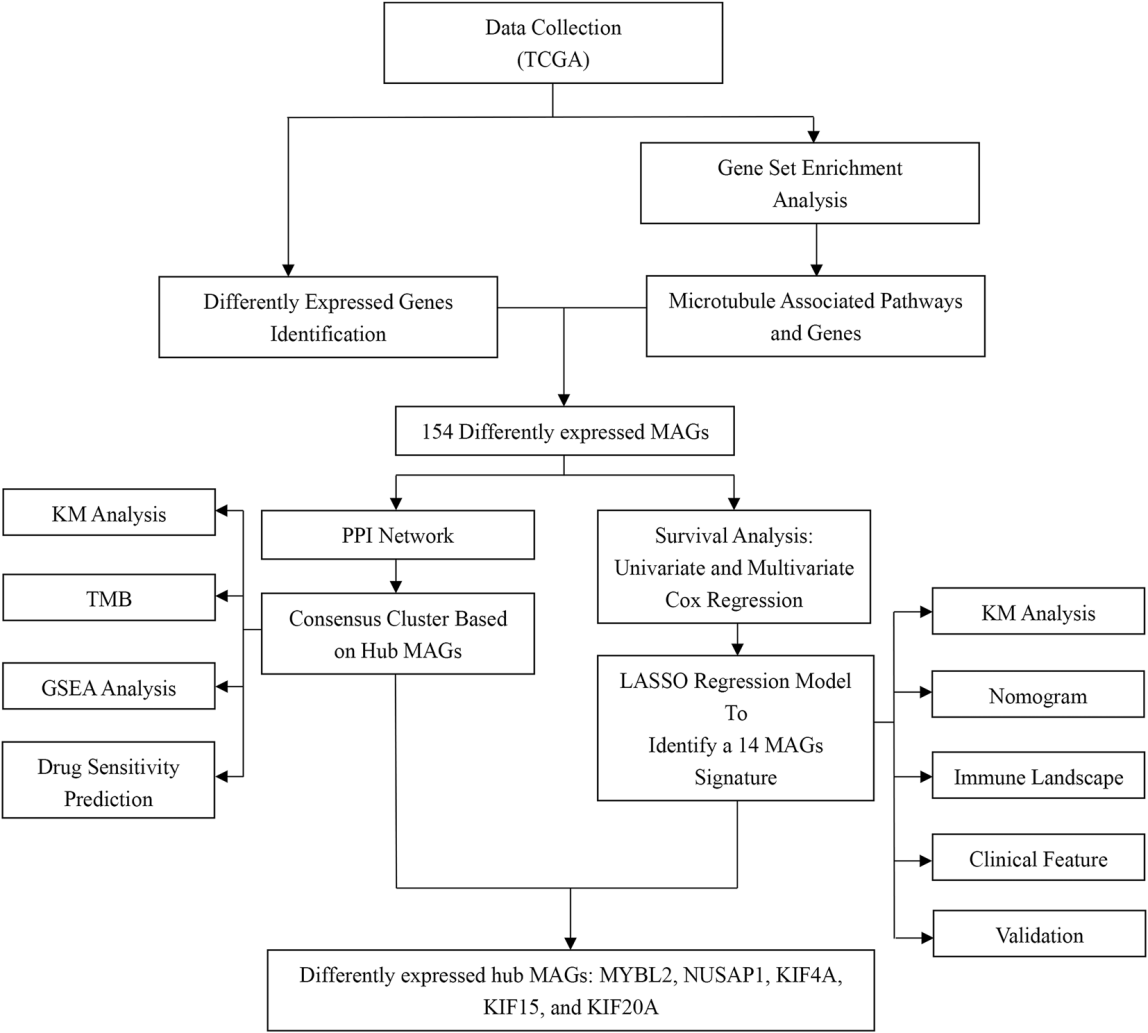
This is the workflow visualization of this research.

**Figure 2:**
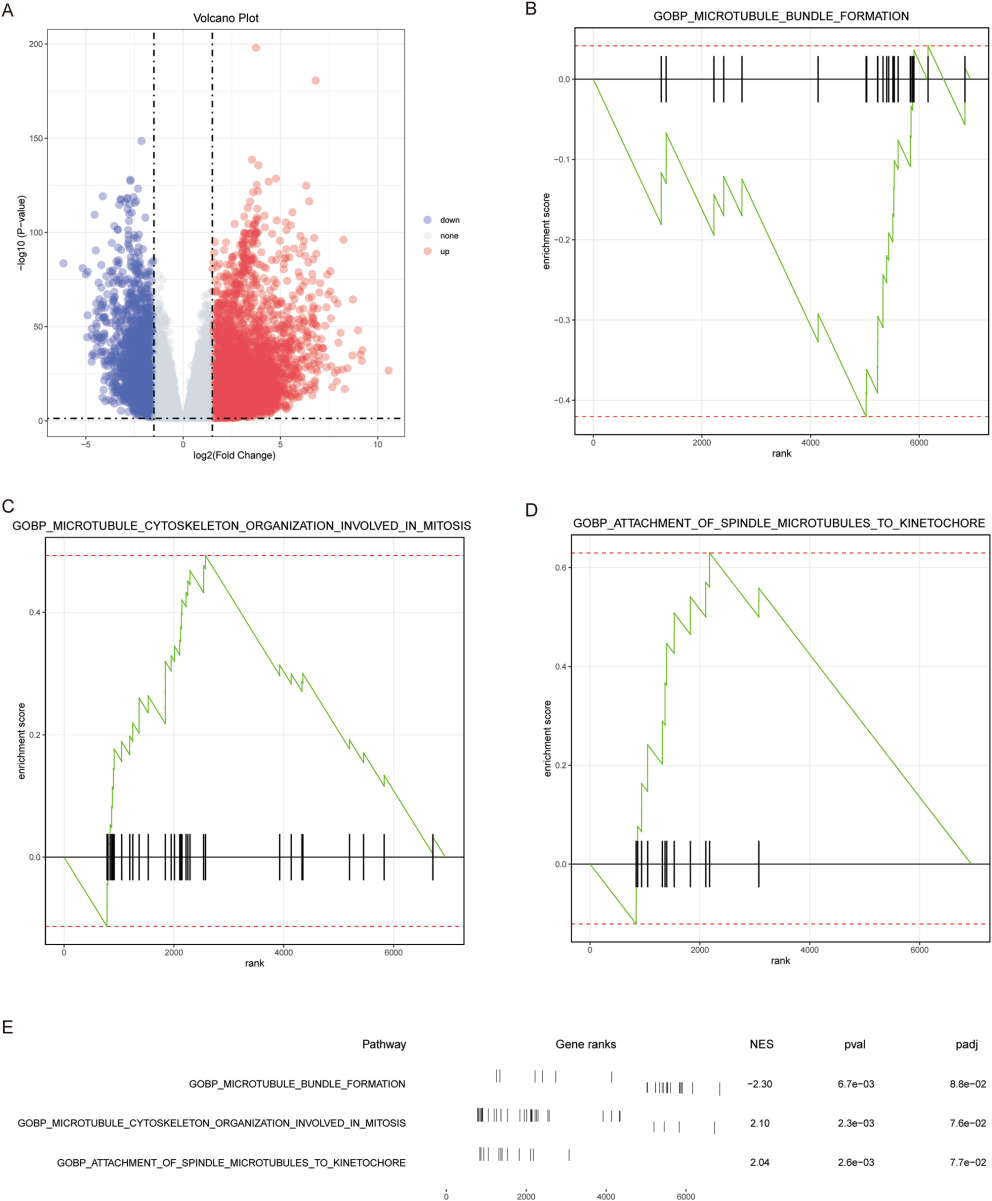
Genes and pathways differentially expressed in LUAD. (**A**) DEGs in the training cohort (n = 489). (**B**–**D**) Differently expressed microtubule-related pathways. (**E**) A merged plot of GSEA analysis. LUAD: lung adenocarcinoma; DEGs: differently expressed genes; GSEA: gene set enrichment analysis.

The diagram of the experiment workflow is presented in Fig. 1. After difference-in-difference analysis, 6940 genes (FC > 1.5, P < 0.05) were obtained. DEGs were displayed on the volcano map (Fig. 2A). Here, GSEA analysis was performed to explore the biological processes involved in tumorigenesis. Surprisingly, the GSEA analysis revealed three microtubule-related pathways as follows: GOBP_MICROTUBULE_BUNDLE_FORMATION, GOBP_MICROTUBULE_CYTOSKELETON_ORGANIZATION_INVOLVED_IN_MITOSIS and GOBP_ATTACHMENT_OF_SPINDLE_MICROTUBULES_TO_KINETOCHORE. GOBP_MICROTUBULE_BUNDLE_FORMATION was significantly enriched in normal samples, while others were significantly enriched in the tumor. Details of pathways are displayed in Fig. 2B–E. The role of microtubules in LUAD was highlighted and worthy of future investigation.

By searching in the MSigDB database, 30 microtubule-related ontology gene sets were discovered. After deduplication, 956 genes that participated in these pathways were considered MAGs. After the intersection of DEGs and MAGs was taken, 154 differently expressed MAGs were selected for further analysis.

### Construction of microtubule-associated prognostic signature

**Figure 3:**
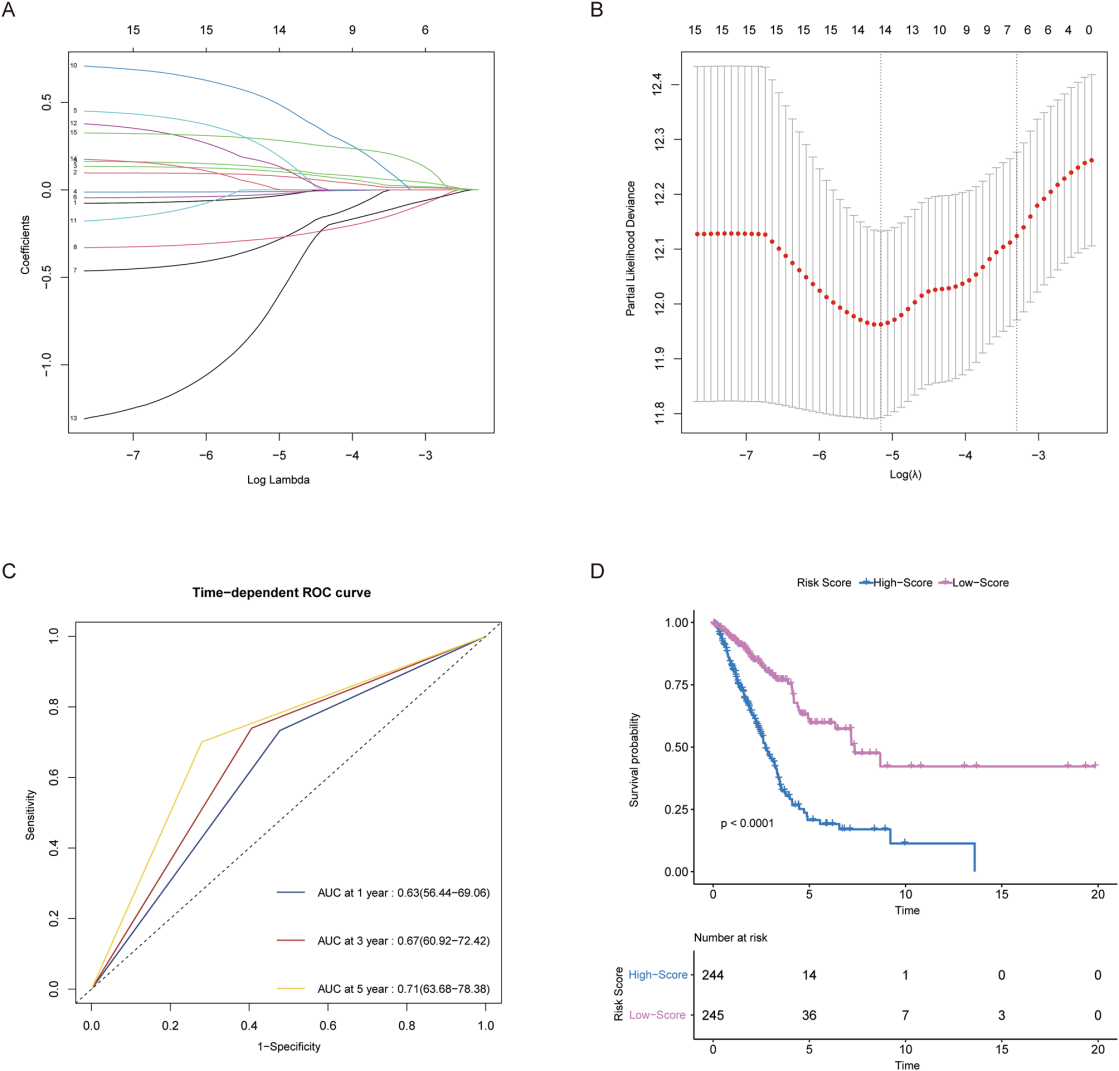
Construction of microtubule-associated prognostic signature. (**A**,**B**) LASSO analysis discovered MAGs for constructing a signature. (**C**) Time-dependent ROC curves and AUC. (**D**) Results of the KM analysis of overall survival for low- and high-score groups. LASSO: the least absolute shrinkage and selection operator; MAGs: microtubule-associated genes; ROC: receiver operating characteristic; AUC: area under the curve; KM analysis: Kaplan–Meier analysis.

A total of 15 genes were identified as independent prognostic MAGs and selected for the building of prognostic models. Then, 14 genes were screened by LASSO regression to build a microtubule-associated risk signature (Fig. 3A,B). The formula is as follows:

$$ \begin{aligned} {\text{Riskscore }} & = \, \left( { - 0.0{39459}0{74148}0{11}*{\text{TUBB3}}} \right) \, + \, \left( {0.0{826175}0{98637292}*{\text{KIF4A}}} \right) \, \hfill \\  & \quad + \, \left( {0.{1}0{91}0{4945255747}*{\text{KIF2}}0{\text{A}}} \right) \, + \, \left( { - 0.0{1}00{318589132996}*{\text{MYBL2}}} \right) \, \hfill \\& \quad + \, \left( {0.{234398648}0{2325}*{\text{SGO1}}} \right) \, + \, \left( { - 0.0{262}0{4222}0{273366}*{\text{NUSAP1}}} \right) \, \hfill \\ & \quad + \, \left( { - 0.{31}0{922864156712}*{\text{KIF15}}} \right) \, + \, \left( { - 0.{2787599}0{8343141}*{\text{NCKAP5}}} \right) \, \hfill \\ & \quad + \, \left( {0.{1284164945}0{23}0{7}*{\text{CDK5R1}}} \right) \, + \, \left( {0.{519865}0{7194}0{523}*{\text{FSIP2}}} \right) \, \hfill \\ & \quad + \, \left( {0.{155416212889263}*{\text{DNAI2}}} \right) \, + \, \left( { - 0.{7}0{3695256275}0{53}*{\text{DNAH12}}} \right) \, \hfill \\ & \quad + \, \left( {0.0{193141341488732}*{\text{ZBBX}}} \right) \, + \, \left( {0.{28855536}0{69112}*{\text{NLRP5}}} \right) \hfill \\ \end{aligned} $$

Then, the area under the ROC curve (AUC) was calculated to preliminarily test efficacy. Time-dependent curves are shown in Fig. 3C, which demonstrated strong predictive capacity for 1, 3 and 5 years of survival (AUC = 0.63 for 1 year of survival, 0.67 for 3 years of survival and 0.67 for 5 years of survival).

### Establishment and evaluation of a nomogram

**Figure 4:**
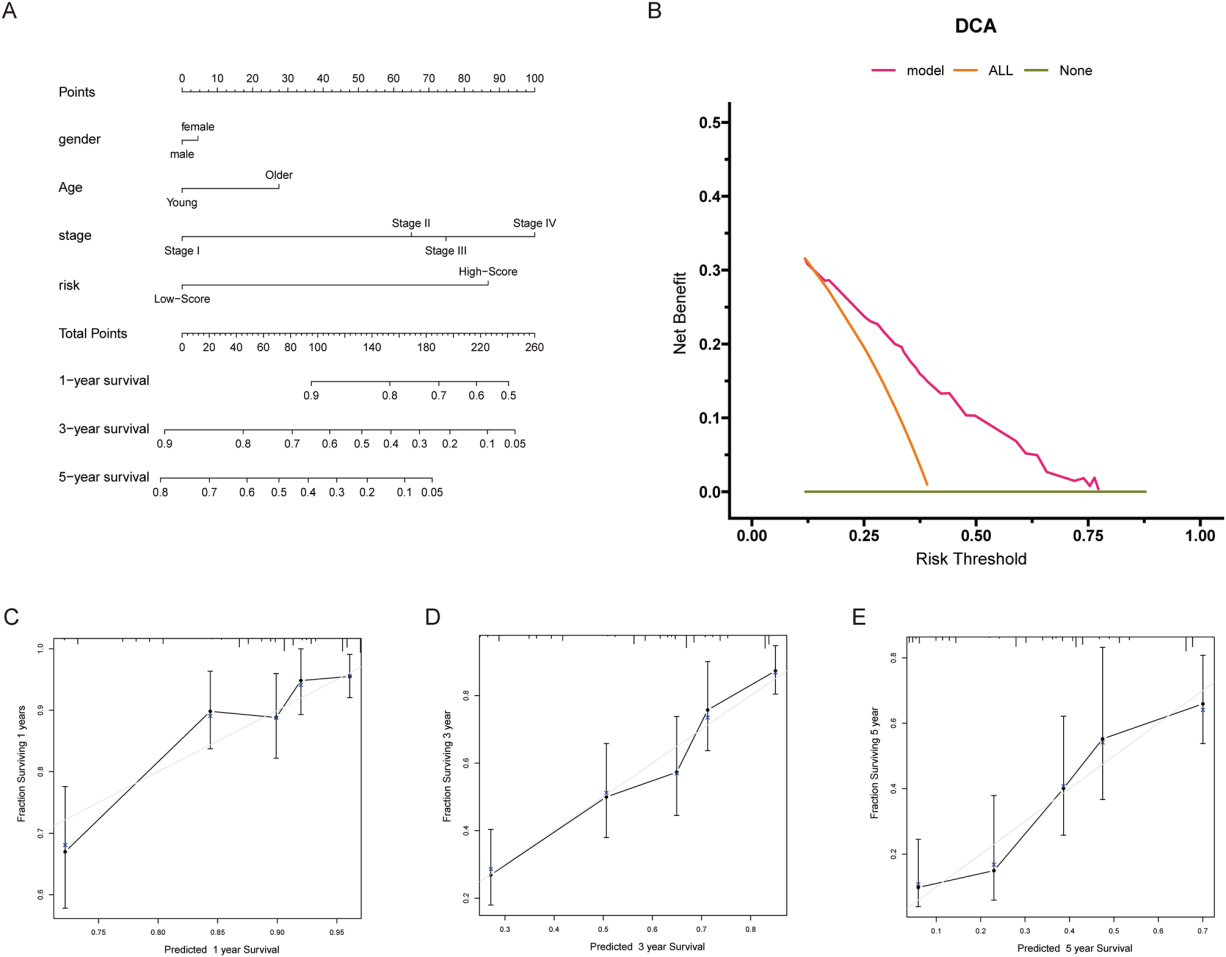
Establishment and evaluation of a nomogram. (**A**) Nomogram plot for predicting 1, 3 and 5-year overall survival. (**B**) DCA curve used for evaluating discrimination ability. (**C**–**E**) Calibration curves for 1, 3 and 5 years to testify uniformity power. DCA: decision curve analysis.

In Fig. 4A, a nomogram that includes four parameters (gender, age, stage and signature risk) was established. Then, several quantitative metrics were used to evaluate the signature, including c-index, and ROC and DCA curves. The c-index, which ranges between 0.5 and 1, is a regularly used metric for signature prediction accuracy. The signature provided a c-index of 0.717, which indicated its great performance in prediction accuracy. DCA curves are a simple method of measuring the clinical utility of different models. As seen in DCA curves, the curve representing the signature did not cross the two extreme curves, which also demonstrated a significant level of net benefit over a broad range of risk thresholds. As a result, the signature may provide clinical benefits for patients (Fig. 4B). All of the above metrics indicated adequate discriminatory capacity. According to the calibration curves of 1, 3 and 5 years, the calculated and actual parameters were near on plots (Fig. 4C–E), which confirmed the effectiveness of the signature in prediction consistency.

### Worse clinical outcomes of the high-score group

After the classification of patients by median risk scores, KM analysis revealed a significant difference between the two groups. The high-score group experienced a faster reduction in survival chances over time (Fig. 3D).

To broaden the applicable scope of the signature, the relationship between the signature and clinical traits was explored. According to the results of clinical analysis, significant differences were found between groups in N (P = 0.03) and stage (P = 0.046). Patients with higher risk scores are prone to develop node metastasis and have a higher stage (Table 1).

**Table 1:** Clinical features of high- and low-score groups.

		High-score	Low-score	P
Sample		244	245	
Gender	Female (%)	123 (50.4)	141 (57.6)	0.135
Age	Age < 60 (%)	85 (35.4)	67 (28.0)	0.101
M (%)	M0	172 (70.5)	154 (62.9)	0.348
	M1	11 (4.5)	6 (2.4)	
	M1a	0 (0.0)	2 (0.8)	
	M1b	3 (1.2)	2 (0.8)	
	MX	58 (23.8)	81 (33.1)	
N (%)	N0	145 (59.4)	173 (70.6)	0.030
	N1	52 (21.3)	40 (16.3)	
	N2	41 (16.8)	27 (11.0)	
	N3	2 (0.8)	0 (0.0)	
	NX	4 (1.6)	5 (2.0)	
T (%)	T1	27 (11.1)	37 (15.1)	0.334
	T1a	19 (7.8)	27 (11.0)	
	T1b	25 (10.2)	30 (12.2)	
	T2	82 (33.6)	76 (31.0)	
	T2a	40 (16.4)	39 (15.9)	
	T2b	14 (5.7)	9 (3.7)	
	T3	24 (9.8)	20 (8.2)	
	T4	12 (4.9)	5 (2.0)	
	TX	1 (0.4)	2 (0.8)	
Stage (%)	Stage I	3 (1.2)	2 (0.8)	0.046
	Stage IA	49 (20.1)	79 (32.2)	
	Stage IB	67 (27.5)	66 (26.9)	
	Stage II	1 (0.4)	0 (0.0)	
	Stage IIA	24 (9.8)	26 (10.6)	
	Stage IIB	36 (14.8)	32 (13.1)	
	Stage IIIA	42 (17.2)	27 (11.0)	
	Stage IIIB	8 (3.3)	2 (0.8)	
	Stage IV	14 (5.7)	11 (4.5)	

### Immune landscape comparison

**Figure 5:**
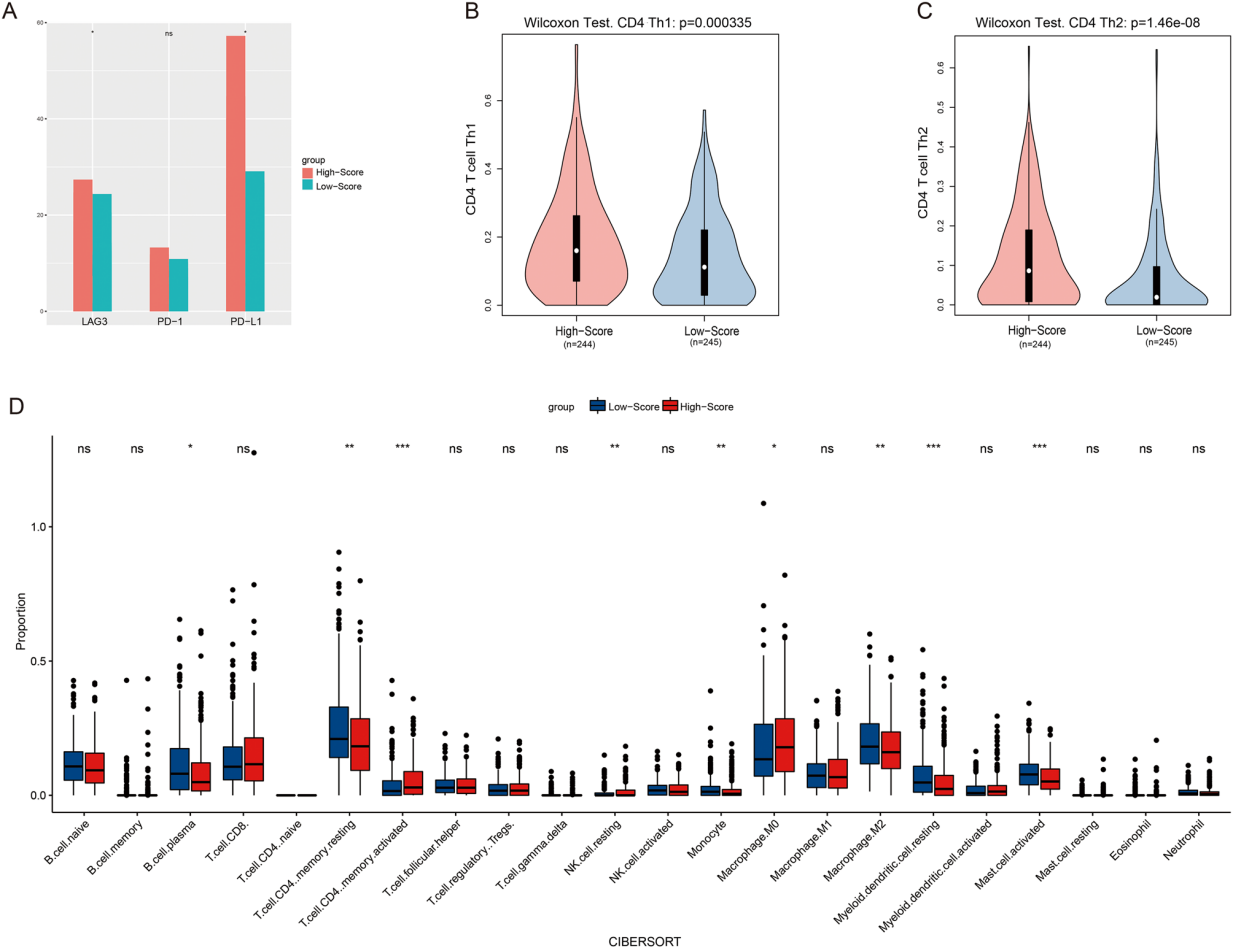
Comparison of the immune landscape between high- (n = 244) and low-score groups (n = 245) in the training cohort. (**A**) Immune checkpoint expression levels. (**B**) Infiltration of CD4+ Th1 cells estimated by xcell. (**C**) Infiltration of CD4+ Th2 cells estimated by xcell. (**D**) Infiltration of 22 common immune cells estimated by cibersort.

The high-score group had higher expression levels of PD-L1 and LAG3 compared with the low-score one (Fig. 5A). Immunotherapy has a greater possibility of helping patients with greater immune checkpoint levels. It indicated that patients in the high-score group may benefit from ICIs. Proportions of the cluster of CD4+ T Th1 and CD4+ Th2 cells were estimated by xcell and depicted in Fig. 5B,C. The immune infiltration heterogeneity of the two groups was investigated using the CIBERSORTABS algorithm (Fig. 5D). With the exception of helper T, mast and natural killer (NK) cells, as well as macrophages, most immune cells showed no significant difference. The high score group was more abundant in M0 macrophages and resting NK, CD4+ Th1 and CD4+ Th2 cells. Mast cells and M2 macrophages were observed to infiltrate more in the low-score group. Immune infiltration imbalance may be involved in the prognostic difference between low- and high-score groups. It may affect the response of ICIs by influencing the expression of immune checkpoints.

### External validation of prognosis and immune characteristics

**Figure 6:**
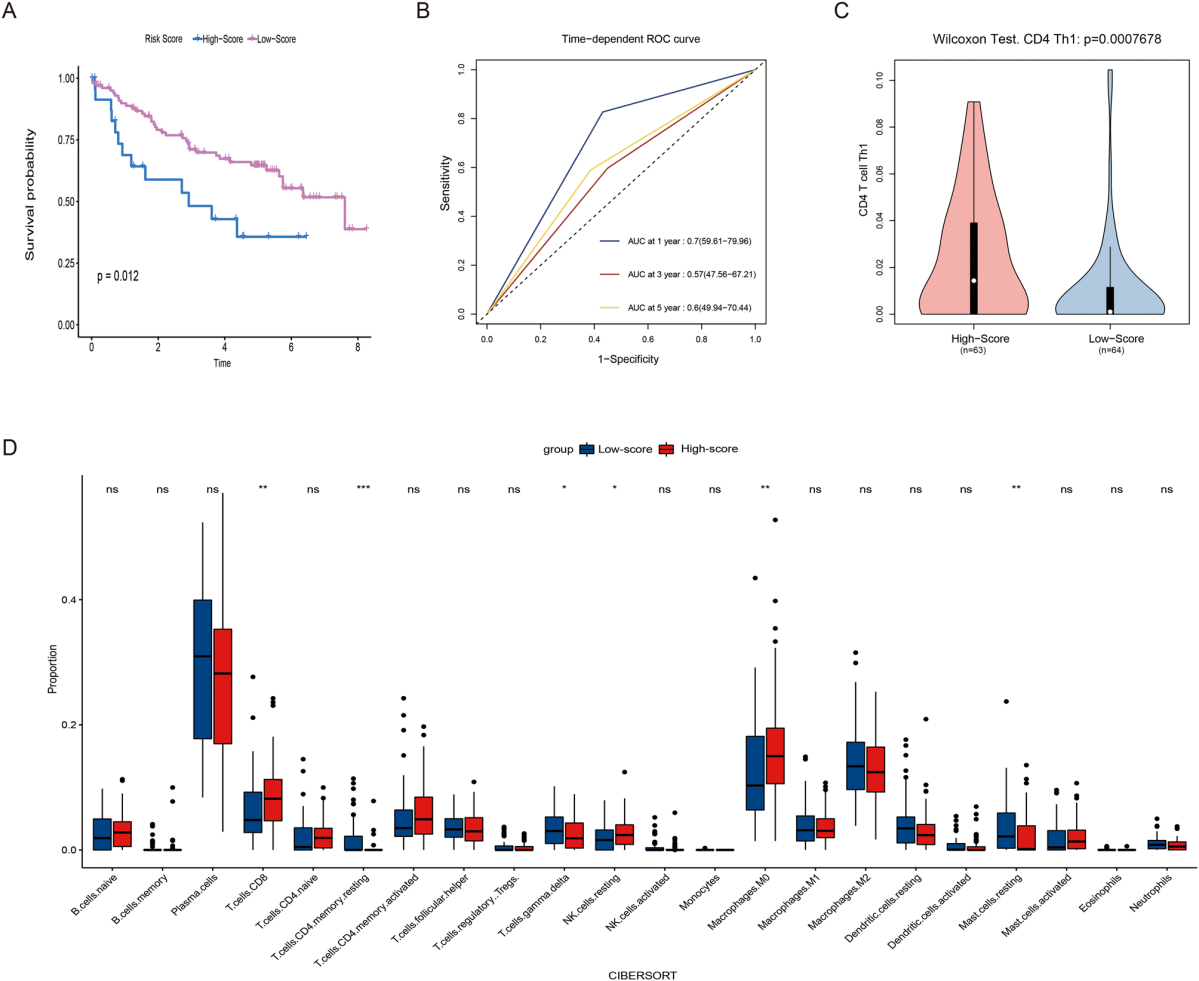
External validation of the signature in GSE50081 (n = 127). (**A**) Results of the KM analysis of overall survival for low- and high-score groups. (**B**) Time-dependent ROC curves and AUC. (**C**) Infiltration of CD4+ Th1 cells estimated by xcell. (**D**) Infiltration of 22 common immune cells estimated by cibersort.

In the external validation set, the risk scores of each patient were calculated and then divided into low- and high-score groups. KM analysis showed that the high-score group reported a worse prognosis (Fig. 6A). With AUC up to 0.7, time-dependent ROC curves indicated excellent predictive ability (Fig. 6B). The results of immune infiltration were brief to those in the TCGA collection. The high-score group demonstrated a higher level of CD4+ Th1 and NK cells, as well as M0 macrophages compared with the low-score one (Figs. 6C,D).

### PPI network of differently expressed MAGs

**Figure 7:**
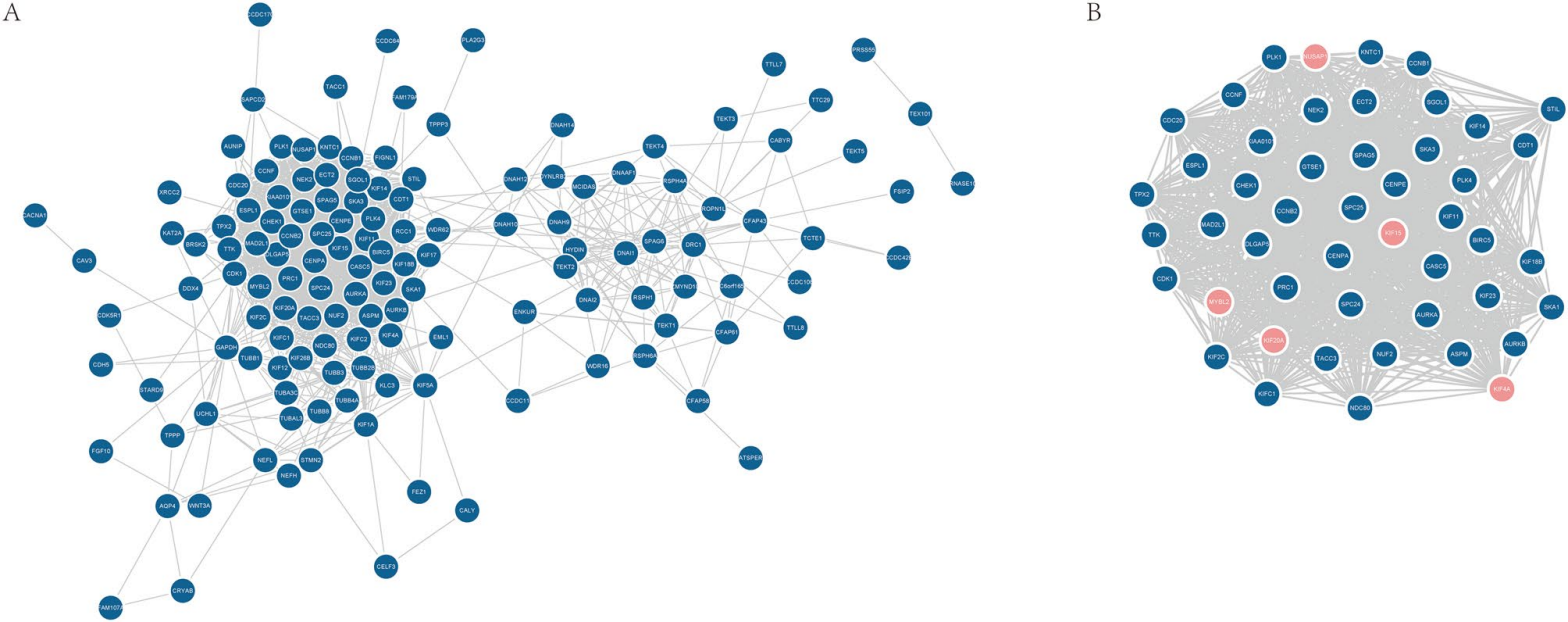
(**A**) PPI network of 154 differently expressed MAGs. (**B**) 48 microtubule-associated proteins screened by MCODE. MCODE: molecular complex detection.

Based on 154 differently expressed MAGs, corresponding encoding proteins were obtained. The PPI network of 154 proteins is depicted in Fig. 7A. Then, subnetworks were identified from the network (Fig. 7B). A total of 48 proteins in the subnetworks had close connections with others. Corresponding to 48 proteins, 45 differently expressed MAGs were considered hub genes in the microtubule-associated subnetwork in LUAD. A total of five hub genes were incorporated into the prognostic signature, including KIF4A, KIF15, KIF20A, MYBL2 and NUSAP1.

### Identification of subtypes based on MAGs

**Figure 8:**
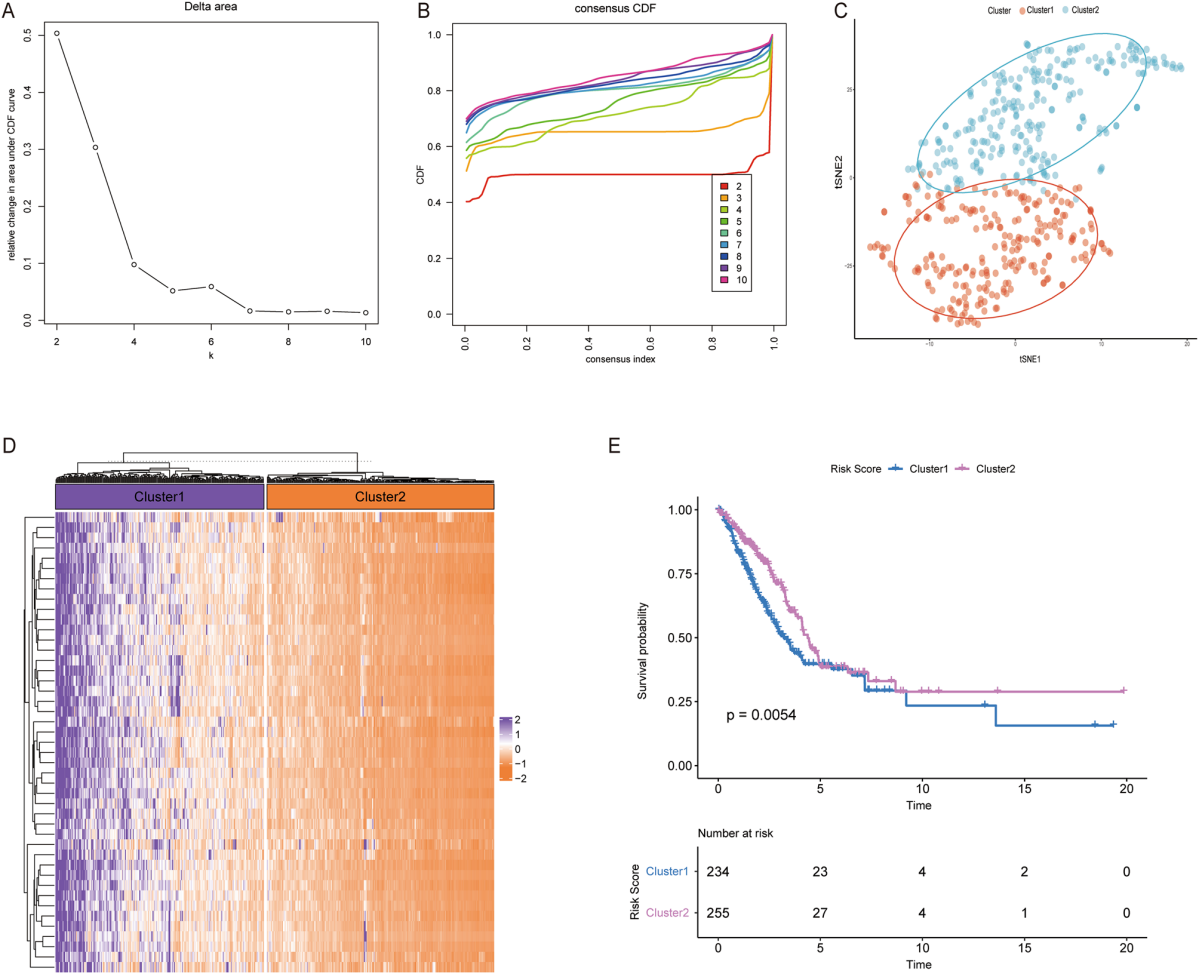
Identification of subgroups using consensus clustering. (**A**) Changes in the area under the CDF curve. (**B**) CDF curve. (**C**) tSNE analysis of clusters. (**D**) Heatmap of clusters. (**E**) KM analysis between clusters. CDF: consensus clustering distribution function.

To learn more about the biological function of 45 MAGs, unsupervised clustering was performed based on their expression. According to the findings, k = 2 was the optimal number of clusters (Fig. 8A,B). Sample distribution was the most stable when the samples were split into two clusters. Then, a tSNE analysis was run on it. A total of 489 patients were divided into two clusters with significant between-group variations in tSNE analysis, which verified the classification efficiency of clustering (Fig. 8C). A heatmap was drawn based on the expression profiles of 45 MAGs. According to the heatmap, the expression of 45 MAGs remained at a relatively high level in cluster1 and a lower level in cluster2 (Fig. 8D). Then, KM analysis was performed to explore the impact of 45 MAGs on survival. It indicated that patients in cluster1 had worse overall survival (Fig. 8E).

### Biological pathways and therapeutic effects

**Figure 9:**
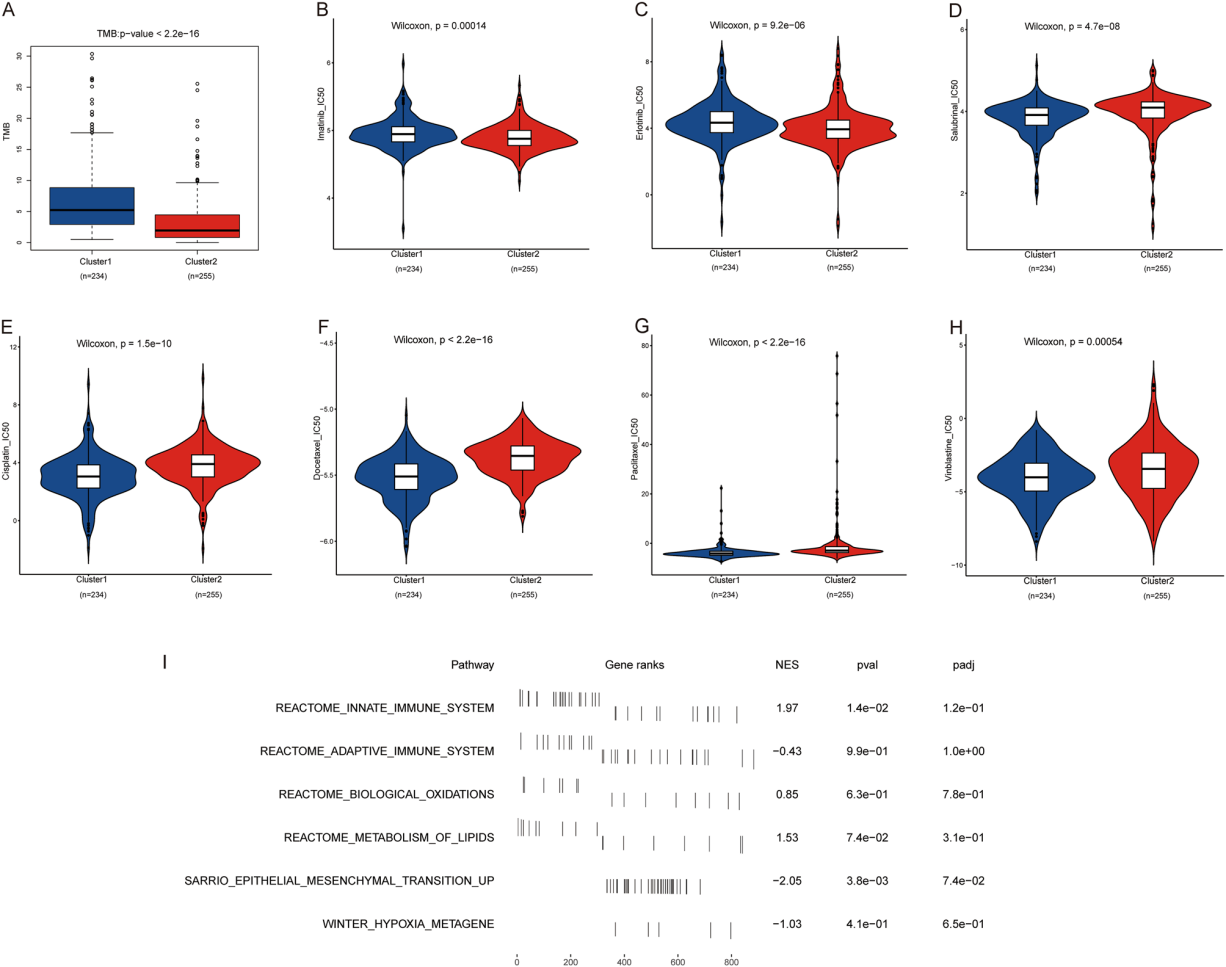
Comparison of biological pathways and therapeutic effects between clusters. (**A**) TMB values in two clusters. (**B**–**D**) Comparison of IC50 in Imatinib (**B**), Erlotinib (**C**) and Salubrinal (**D**). (**E**–**H**) Comparison of IC50 in Cisplatin (**E**), Docetaxel (**F**), Paclitaxel (**G**) and Vinblastine (**H**).

TMB was assessed as a measure of immunotherapy response. With more mutation-derived antigens, T cells tend to recognize neoantigens and kill cancerous cells^16^. As a widely accepted immunotherapy biomarker, patients with higher TMB are more inclined to gain more benefits from ICB therapy. Patients in cluster1 had significantly higher TMB than those in cluster2, which indicated that immunotherapy would benefit them more (Fig. 9A). The IC50 of commonly used chemotherapeutic drugs is shown in Fig. 9E–H. Patients in cluster1 had a lower IC50 of chemotherapeutic drugs incorporating cisplatin, docetaxel, paclitaxel and vinblastine, which indicated better chemosensitivity. IC50 values varied depending on the targeted medicines (Fig. 9B–D). Patients with a high expression level of hub MAGs may find it difficult to benefit from erlotinib. Erlotinib is used for advanced non-small cell lung cancer (NSCLC) that has failed chemotherapy. On the other hand, salubrinal would more benefit hub MAGs.

As indicated by the GSEA analysis, hub MAGs were involved in multiple oncogenic pathways. Microtubule-associated pathways were the top-ranked pathways in the GSEA-enriched pathways, which confirmed the association between the functions of hub MAGs and microtubules. Besides, other cancer-associated pathways were also found in the GSEA-enriched pathways. Some of the enrichment results are shown in Fig. 9I. Except for microtubule-associated pathways, hub MAGs are also engaged in other critical pathways including immune, oxidations, metabolism, EMT and hypoxia. They may be implicated in other cancer-related pathways to promote the malignant phenotype of cancer.

### Verification of differently expressed hub MAGs

**Figure 10:**
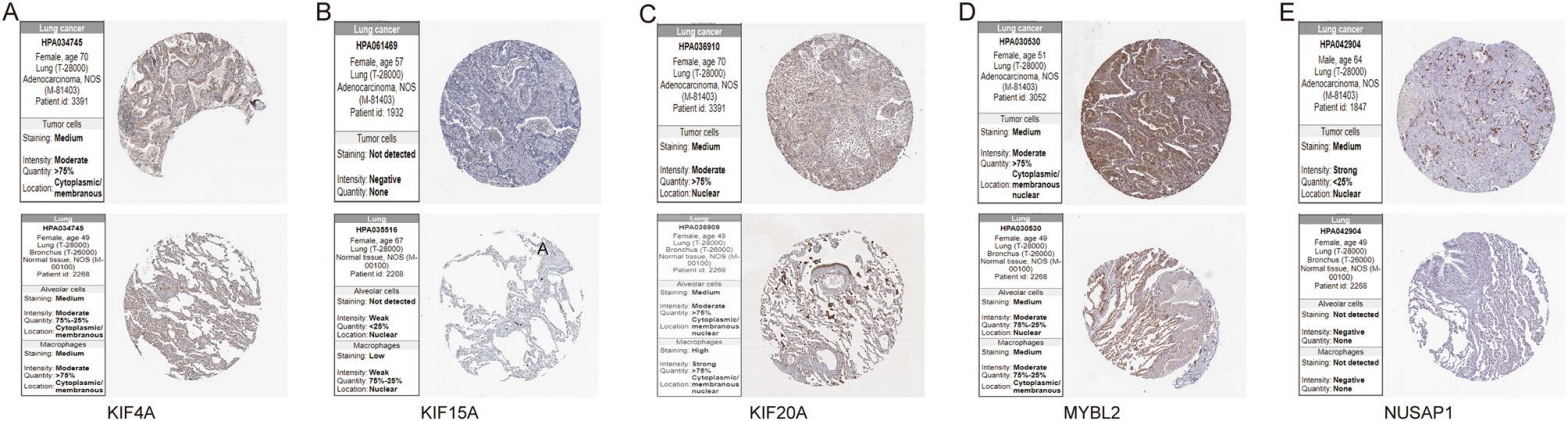
Immunohistochemical analysis of five MAGs in the HPA database. (**A**) KIF4A. (**B**) KIF15. (**C**) KIF20A. (**D**) MYBL2. (**E**) NUSAP1.

**Figure 11:**
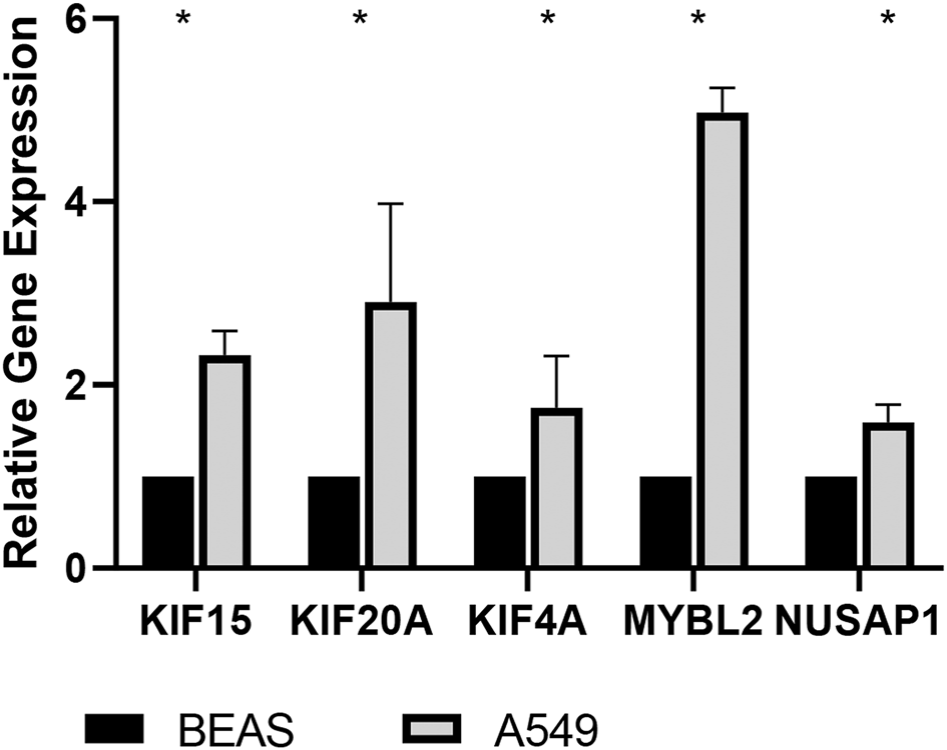
Validation by qRT-PCR. The relative expression levels of five MAGs were detected by qRT-PCR in A549 and BEAS-2B cells. N = 3. * represents P < 0.05. ** represents P < 0.01.

The expression of differentially expressed hub MAGs was verified in the HPA database and experiment. It was shown that all of the five genes were significantly overexpressed in LUAD (Fig. 10A–E). Compared with BEAS-2B cells, they experienced a significant increase in A549 cells (Fig. 11).

## Discussion

In this research, it was found that microtubule-associated biological processes were differently expressed significantly between tumor and normal samples. Microtubules are a well-known anti-cancer target. However, the clinical application of MTAs is restricted by toxicity and drug resistance. The exploration of novel molecular targets with high efficiency is necessary. Previous research mainly focused on a single gene, but little literature described the characteristics of all MAGs. Singharajkomron^17^ investigated the prognostic function of microtubule-associated proteins in lung cancer and distinguished six genes as biomarkers. Consistent with this, KIF4A was also identified as a biomarker in this research. However, their methods of subsequent analysis mainly focused on prognostic value of a single gene. In contrast, this study was more inclined to make an overall analysis of MAGs. In addition, the value of MAGs in guiding treatment was further assessed in this research.

Several genes in the model had already been proven as independent prognostic factors. Tubulin β 3 (TUBB3) was overexpressed in various cancers and linked to the poor response of MTAs^18^. It has been discovered to be involved in the postoperative survival of NSCLC patients^19^.

In general, patients with high scores developed more advanced clinical stages and were more likely to develop lymphatic metastasis. Cancer metastasis is a complex process which involves both tumor cell phenotype and the tumor microenvironment (TME)^20^. Lymph node and blood metastasis are the most common metastatic routes. Tubulin, a part of the cytoskeleton, works with actin to create impetus through contraction. Depending on this physical process, microtubules promote tumor metastasis through cellular division, cell shape and migration^21^. It has been discovered that TUBB3 inhibits migration by interfering with microtubule dynamics in malignant melanoma cells^22^. It could explain the malignant phenotype of the high-score group.

Immunotherapy is an emerging and promising therapy for NSCLC patients. However, the relationships between the immune microenvironment and microtubules still require further investigation. As complex surroundings that consist of immune and intestinal cells, TME exerts an influence on tumor regression and therapeutic response^23,24^. Tumor immune infiltration differed between low- and high-score groups, which was validated in the external cohort. Patients with high-risk scores demonstrated a higher infiltration of resting NK and activated CD4+ T cells. NK cells are the only innate immune cells to kill tumor cells directly. Resting and activating NK cells are the adverse status of NK cells. The activation of resting NK cells is regulated more strictly with less cytotoxicity^25^. Given the higher infiltration of resting NK cells in the high-score group, immunotherapy based on the activation of NK cells might be a promising therapy. CD4+ T cells are perceived to exert an anti-tumor effect mainly by helping CD8+ cytotoxic T lymphocytes. Other inhibitor factors may account for no significant difference in CD8+ T cell counts between groups.

The comparison of immune checkpoint expression levels showed that the high-score group expressed higher levels of PD-L1 and LAG3. This indicates that patients with a high score may benefit from immunotherapy.

Based on 154 differently expressed MAGs, hub genes were screened by a PPI network. Then, two distinct molecular subtypes were identified based on the expression characteristics of 45 hub MAGs. The survival outcomes and therapy responses of these two subgroups differed significantly.

GSEA analysis revealed that hub MAGs may be involved in multiple oncogenic pathways. Su^26^ discovered that the protein regulator of cytokinesis 1 (PRC1) mediated the recruitment of tumor-associated macrophages and regulatory cells (Tregs), which facilitated immunosuppression and angiogenesis in double-negative prostate cancer. Xu^27^ reported that the overexpression of epithelial cell transforming sequence 2 (ECT2) would promote the polarization of M2 macrophages in hepatocellular carcinoma. It has been identified that TPX2 level is correlated with EMT in hepatocellular carcinoma and cholangiocarcinoma^28,29^. STIL has been reported recently to enhance metastasis in lung cancer by EMT and hypoxia^30^.

Then, the relationship between hub MAGs and various therapeutic responses was explored. Patients in cluster1 would benefit from immunotherapy and chemotherapy. On the contrary, patients in cluster2 seemed more suitable for targeted therapy. Hub MAGs are involved in not only chemotherapy sensitivity but also immunotherapy response and targeted therapy, which may serve as a reference for therapy selection.

Surprisingly, five genes were included in both the prognostic signature and hub MAGs. They are not only critical in the aberrant activities of microtubules but also likely to be involved in other carcinogenic processes. Hence, the five genes might be potential prognostic factors and therapeutic targets.

The pan-cancer analysis indicated that MYBL2 was observed to be an overexpressed oncogene in multiple cancers. The underlying mechanism might include the regulation of the immune microenvironment and cell proliferation and spread^31^. Its ability to promote cell proliferation has been experimentally verified in NSCLC^32^. It not only promoted cancer metastasis via EMT but also regulated cancer therapy resistance through the prolongation of cell survival^33^. NUSAP1 is involved in microtubule organization and binding, and its promoting effect on cancer malignant phenotype has been explored in multiple studies^34^. The immune infiltration analysis of hepatocellular carcinoma showed that NUSAP1 might regulate the immune microenvironment by influencing CD4+ T cells^35^.

Kinesins are a type of motor protein that moves along microtubules, which supports a large number of essential functions including cell division and transport. It has been found that kinesins play a key role in cancer progression. Their expression has been reported to link to prognosis, immunological response, drug assistance and other oncogene pathways. The importance of kinesins in the mitogen-activated protein kinase (MAPK) pathway has been studied extensively, which is a complex cascade reaction in essential cell activities^36^.

It has been identified that KIF4A, KIF15 and KIF20A as members of the kinesin family are risk factors with prognostic value in LUAD^37–39^. The overexpression of these genes promotes cancer by not just directly controlling tumor cells, but also affecting the tumor immune microenvironment. In bladder cancer, KIF4A potentiated an immunosuppressive microenvironment by increasing the recruitment of myeloid-derived suppressor cells^40^. KIF15 functioned in macrophage polarization, and its decreased expression resulted in the enhancement of macrophage migration^41^. Furthermore, it was worth noting that KIF20A was linked to CD4+ T-cell response, which was consistent with the above-described immune analysis. In immunotherapy-treated patients with head-and-neck malignant tumors, KIF20A-specific Th1-cell responses were observed^42^. In addition, these five genes were reported to be involved in multiple oncogenic pathways. KIF4A, for example, was observed to accelerate angiogenesis in renal cell carcinoma cells^43^ and enhance drug resistance in LUAD^44^.

To summarize, these five genes were closely related to multiple cancers. They were key factors in tumor progression and influenced it both directly and indirectly. These five genes might be effective therapeutic targets in the future.

As is known to all, little literature has discussed the prognostic value of microtubules before. It is the first comprehensive analysis of MAGs in LUAD. The findings may provide new insight into the role of microtubules in the progression, prognosis and treatment of LUAD. Of course, limitations also exist in this research. The expression of hub MAGs in cells was verified. Further validation in clinical samples and further mechanistic investigation are needed.

## Conclusion

A microtubule-related prognosis signature which can serve as an independent prognostic factor was constructed. MAGs were proved to be important factors that regulated therapy response. Five key MAGs were verified as novel prognostic markers and therapeutic targets. In this study, the values of MAGs in prognosis and guiding therapy were explored, which provided evidence for the prognosis and treatment of LUAD.

## Data Availability

The datasets presented in this study can be found in the TCGA-LUAD project (https://portal.gdc.cancer.gov) and GSE50081 (https://www.ncbi.nlm.nih.gov/geo/).
